# Inhibitory effect of a tyrosine-fructose Maillard reaction product, 2,4-bis(*p*-hydroxyphenyl)-2-butenal on amyloid-β generation and inflammatory reactions via inhibition of NF-κB and STAT3 activation in cultured astrocytes and microglial BV-2 cells

**DOI:** 10.1186/1742-2094-8-132

**Published:** 2011-10-07

**Authors:** Young-Jung Lee, Dong-Young Choi, Im Seup Choi, Jin-Yi Han, Heon-Sang Jeong, Sang Bae Han, Ki-Wan Oh, Jin Tae Hong

**Affiliations:** 1 College of Pharmacy, Chungbuk National University, 12 Gaesin-dong, Heungduk-gu, Cheongju, Chungbuk 361-763, Korea; 2 Medical Research Center, Chungbuk National University, 12 Gaesin-dong, Heungduk-gu, Cheongju, Chungbuk 361-763, Korea; 3 CBITRC, Chungbuk National University, 12 Gaesin-dong, Heungduk-gu, Cheongju, Chungbuk 361-763, Korea; 4 College of Agriculture, Life and Environments Sciences, Chungbuk National University, 12, Gaeshin-dong, Heungduk-gu, Cheongju, Chungbuk, 361-763, Korea

**Keywords:** 2,4-bis(*p*-hydroxyphenyl)-2-butenal, NF-κB, STAT3, neuroinflammation, amyloidogenesis

## Abstract

**Background:**

Amyloidogenesis is linked to neuroinflammation. The tyrosine-fructose Maillard reaction product, 2,4-bis(*p*-hydroxyphenyl)-2-butenal, possesses anti-inflammatory properties in cultured macrophages, and in an arthritis animal model. Because astrocytes and microglia are responsible for amyloidogenesis and inflammatory reactions in the brain, we investigated the anti-inflammatory and anti-amyloidogenic effects of 2,4-bis(*p*-hydroxyphenyl)-2-butenal in lipopolysaccharide (LPS)-stimulated astrocytes and microglial BV-2 cells.

**Methods:**

Cultured astrocytes and microglial BV-2 cells were treated with LPS (1 μg/ml) for 24 h, in the presence (1, 2, 5 μM) or absence of 2,4-bis(*p*-hydroxyphenyl)-2-butenal, and harvested. We performed molecular biological analyses to determine the levels of inflammatory and amyloid-related proteins and molecules, cytokines, Aβ, and secretases activity. Nuclear factor-kappa B (NF-κB) DNA binding activity was determined using gel mobility shift assays.

**Results:**

We found that 2,4-bis(*p*-hydroxyphenyl)-2-butenal (1, 2, 5 μM) suppresses the expression of inducible nitric oxide synthase (iNOS) and cyclooxygenase-2 (COX-2) as well as the production of nitric oxide (NO), reactive oxygen species (ROS), tumor necrosis factor-α (TNF-α), and interleukin-1β (IL-1β) in LPS (1 μg/ml)-stimulated astrocytes and microglial BV-2 cells. Further, 2,4-bis(*p*-hydroxyphenyl)-2-butenal inhibited the transcriptional and DNA binding activity of NF-κB--a transcription factor that regulates genes involved in neuroinflammation and amyloidogenesis via inhibition of IκB degradation as well as nuclear translocation of p50 and p65. Consistent with the inhibitory effect on inflammatory reactions, 2,4-bis(*p*-hydroxyphenyl)-2-butenal inhibited LPS-elevated Aβ_42 _levels through attenuation of β- and γ-secretase activities. Moreover, studies using signal transducer and activator of transcription 3 (STAT3) siRNA and a pharmacological inhibitor showed that 2,4-bis(*p*-hydroxyphenyl)-2-butenal inhibits LPS-induced activation of STAT3.

**Conclusions:**

These results indicate that 2,4-bis(*p*-hydroxyphenyl)-2-butenal inhibits neuroinflammatory reactions and amyloidogenesis through inhibition of NF-κB and STAT3 activation, and suggest that 2,4-bis(*p*-hydroxyphenyl)-2-butenal may be useful for the treatment of neuroinflammatory diseases like Alzheimer's disease.

## Background

Alzheimer's disease (AD) is an age-related neurodegenerative disease characterized by the accumulation of beta amyloid (Aβ), an insoluble peptide deposited extracellularly in the brain, causing senile plaques [1]. This hydrophobic polypeptide is the product of proteolytic cleavage of the amyloid precursor protein (APP). Brains of patients with AD exhibit a number of pathological abnormalities, including a profound loss of synapses, microglial activation, and inflammatory processes [2]. Studies performed in transgenic animals suggest that inflammation plays an important role in the process of cerebral amyloid deposition [3,4]. Inflammatory reactions and mediators have been reported to augment APP expression and Aβ formation [5,6] and transcriptionally upregulate mRNA and protein levels and enzymatic activity of β-secretase, a key enzyme in the production of Aβ [7]. Recently we and others have also shown that lipopolysaccharide (LPS), an inducer of inflammation, can influence Aβ deposition [8,9] and that anti-inflammatory agents prevent Aβ deposition in cultured neuronal cells [9-11], as well as in a mouse models of AD [9,12]. Moreover, McGeer and colleagues proposed possible therapeutic effects of anti-inflammatory agents in patients with AD [13]. These observations strongly suggest that neuroinflammation could be an important causative contributor in the development and/or progression of AD, and anti-inflammatory agents could be effective in dimishing the prevalence of AD through reduction of Aβ generation and/or deposition.

Nitric oxide (NO) is a free radical produced by the inducible NO synthase (iNOS) isoform. Prostaglandins (PGs), products of cyclooxygenase (COX) are essential components of the host innate immune and inflammatory responses that may contribute to pathological processes, in particular, neurodegenerative diseases such as multiple sclerosis, Parkinson's disease, and AD [14]. In most neurodegenerative disorders, massive neuronal cell death occurs as a consequence of an uncontrolled neuroinflammatory response, where activated astrocytes and microglia, together with their cytotoxic agents, play a crucial pathological role [15]. Glial cells, consisting of astrocytes and microglia, can produce cytokines, reactive oxygen radicals, NO, and PGs, which lead to exaggeration of the disease processes [16]. Expression of genes for inflammatory elements such as iNOS and COX-2, as well as cytokines, can be regulated by activation of nuclear factor-κB (NF-κB). There is one NF-κB DNA consensus sequence within the COX-2 promoter [17], and 2 NF-κB DNA consensus sequences within the iNOS promoter [18], which are responsible for NF-κB DNA-binding activity. Moreover, NF-κB DNA consensus sequences are also located in the promoter of neuronal β-secretase (BACE 1). Dysregulation of NF-κB, thus, is associated with many inflammation-associated diseases, as well as the generation of Aβ, implying that appropriate regulation and control of NF-κB activity would provide a potential approach for the management of AD, through the reduction of both neuroinflammation and Aβ generation [19]. Signal transducer and activator of transcription 3 (STAT3) is also a significant regulator of neuroinflammation, Aβ generation [20], and cytokine-driven NF-κB-mediated Aβ gene expression [21].

The Maillard reaction (MR), a well-known, non-enzymatic browning reaction, can produce colored or colorless products from substrates such as glucose-tyrosine, glucose-lysine, fructose-lysine, ribose-lysine, xylose-arginine, xylose-glycine, and xylose-tryptophan [22-25]. These products have anti-oxidative [22-24,26], anti-mutagenic [27], anti-carcinogenic [28] and anti-bacterial effects [29]. Previous studies have shown that LPS treatment of cultured astrocytes causes Aβ accumulation through elevation of β- and γ-secretase activity and inflammatory reactions [9]. We have shown that 2,4-bis(*p*-hydroxyphenyl)-2-butenal inhibits LPS-elevated inflammatory reactions in macrophages (unpublished data). Therefore, in the present study, we investigated whether 2,4-bis(*p*-hydroxyphenyl)-2-butenal inhibits LPS-elevated Aβ levels in cultured astrocytes and microglial BV-2 cells, through attenuation of LPS-induced inflammatory reactions, and investigated possible mechanisms of anti-amyloidogenesis.

## Methods

### Chemicals and reagents

**Figure 1:**
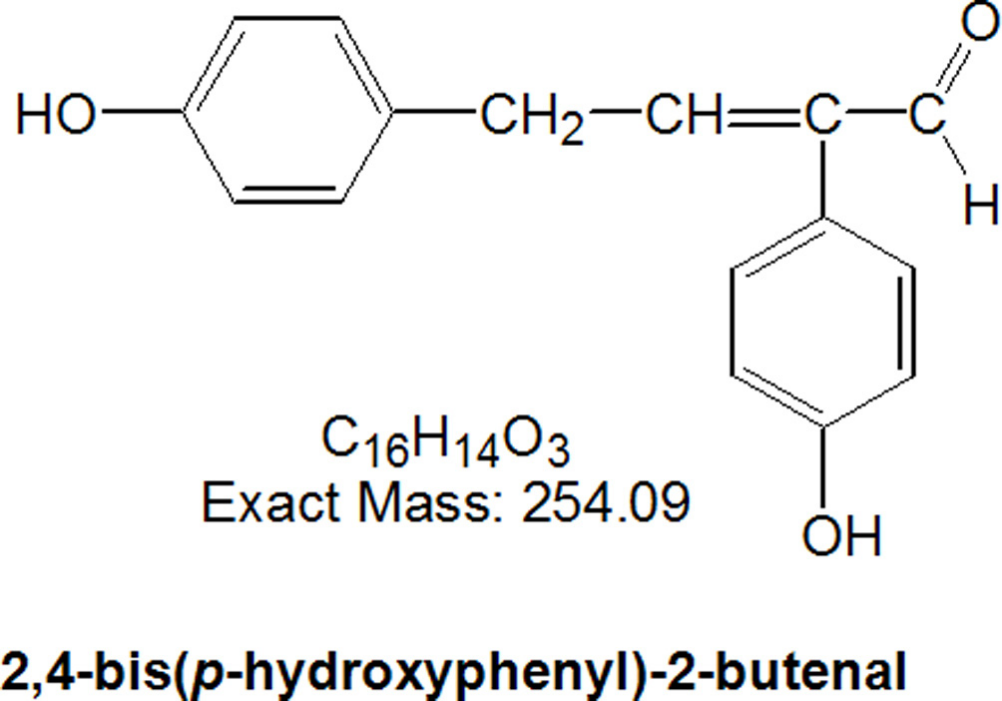
**Chemical structure of 2,4-bis(*p*-hydroxyphenyl)-2-butenal**.

LPS (from *Escherichia coli *055:B5) was obtained from Sigma Aldrich (St Louis, MO). Dulbecco's modified Eagle's medium (DMEM), fetal bovine serum, penicillin and streptomycin were purchased from Invitrogen (Carlsbad, CA). We used siRNA and AG490 for inhibition of STAT3 signaling pathway. A non-targeting control siRNA was purchased from Bioneer (Daejeon, Korea) and siRNA for STAT3 was purchased from Santa Cruz Biotechnology (Santa Cruz, CA). AG490, a selective JAK_2_-specific inhibitor, selectively inhibits STAT3 phosphorylation. This reagent (Sigma Aldrich) was dissolved in dimethylsulfoxide (DMSO, Sigma Aldrich) to a stock concentration of 50 mM, and was diluted to a final concentration of 50 μM with conventional culture medium just before use. Characterization of 2,4-bis(*p*-hydroxyphenyl)-2-butenal has been described elsewhere [30]. 2,4-Bis(*p*-hydroxyphenyl)-2-butenal was prepared as previously reported [30]. In brief, we prepared 100 ml of fructose-tyrosine mixture including 0.1 M tyrosine and 0.05 M fructose. MR was carried out in a temperature-controlled autoclave apparatus (Jisico, Seoul, South Korea) at 130°C for 2 h. After 2 h heating, reaction mixture was filtered through a 0.45 μm membrane and used to isolate the active compounds through several fractionation steps. The fraction of the highest α-glucosidase inhibitory activity from each set of fractions was subjected to the next fractionation step. Fructose-tyrosine MR products were purified using a series of solvent fractionations; ethyl acetate (EtOAc), n-butanol, and water. The EtOAc fraction was subjected to silica gel column chromatography and eluted with increasing concentrations of methanol (MeOH) in dichloromethane (DCM). An A2 fraction via DCM:MeOH (20:1, v:v) of a first silica gel chromatography was separated to 23 sub-fractions via a second silica gel chromatography. Fractions B14 and B15 from the 23 sub-fractions were further purified by semi-preparative high performance liquid chromatography with a C18 column. The structure is shown in Figure 1. 2,4-Bis(*p*-hydroxyphenyl)-2-butenal was dissolved in 0.01% DMSO, and used at concentrations of 2.5, 5, 10 μg/ml to treat cultured cells.

### Astrocytes and microglial BV-2 cell cultures

Rats were maintained in accordance with the Institutional Animal Care and Use Committee (IACUC) of Laboratory Animal Research Center at Chungbuk National University, Korea (CBNU-144-1001-01). Two-day-old rat pups were ice-anesthetized and decapitated. After the skin was opened and the skull was cut, the brain was then released from the skull cavity. After washing with PBS, the cerebrum was separated from cerebellum and brain stem, and the cerebral hemispheres were separated from each other by gently teasing along the midline fissure with the sharp edge of forceps. The meninges were gently peeled from the individual cortical lobes and the cortices were dissociated by mechanical digestion [using the cell strainer (BD Biosciences, Franklin Lakes, NJ, USA)] with DMEM containing F12 nutrient mixture (F12) (Invitrogen, Carlsbad, CA). The resulting cells were centrifuged (1,500 rpm, 5 min), resuspended in serum-supplemented culture media, and plated into 100 mm dishes. Serum-supplemented culture media was composed of DMEM supplemented with F12, FBS (5%), NaHCO_3 _(40 mM), penicillin (100 units/ml), and streptomycin (100 μg/ml). The cells were incubated in the culture medium in a humidified incubator at 37°C and 5% CO_2 _for 9 days. At confluence (9 days), the flask was subjected to shaking for 16-18 h at 37°C, the cultures were then treated for 48 h with cytosine arabinoside and the medium was replaced with DMEM/F12HAM containing 10% FBS. The monolayer was treated with 1.25% trypsin-EDTA for a short duration after which the cells were dissociated and plated onto uncoated glass coverslips. The astrocyte cultures formed a layer of process-bearing, glial fibrillary acidic protein (GFAP)-positive cells. The purity of astrocyte cultures was assessed by GFAP-immunostaining. In our conditions, we found that over 95% of the cells were astrocytes. The cultured cells were treated with LPS with or without 2,4-bis(*p*-hydroxyphenyl)-2-butenal for 24 h (for western blotting, Aβ levels, and secretase activity determinations) or for 1 h for NF-κB DNA activity and western blotting for relative protein expression, or for 8 h for NF-κB luciferase activity assay. Microglial BV-2 cells were maintained with serum-supplemented culture media of DMEM supplemented with FBS (5%), NaHCO_3 _(40 mM), penicillin (100 units/ml), and streptomycin (100 μg/ml). BV-2 cells were incubated in culture medium in a humidified incubator at 37°C and 5% CO_2_. In a manner similar to the methods for astrocytes, BV-2 cells were treated with LPS with or without 2,4-bis(*p*-hydroxyphenyl)-2-butenal for 24 h (for Aβ level and secretase activity determinations) or for 1 h for NF-κB DNA activity and western blotting for relative protein expression.

### Cell viability assay

Cytotoxicity of 2,4-bis(*p*-hydroxyphenyl)-2-butenal was evaluated using a WST-8 assay (Dojindo Laboratories, Tokyo, Japan). WST-8 [2-(2-methoxy-4-nitrophenyl)-3-(4-nitrophenyl)-5-(2,4-disulfophenyl)-2H-tetrazolium, monosodium salt] is reduced by dehydrogenases to give a yellow-colored soluble product (formazan) in the culture medium. The amount of the formazan dye generated is directly proportional to the number of living cells. In brief, 1 × 10^4 ^cells per well were plated into 96-well plates, incubated at 37°C for 24 h, and given a fresh change of medium. Cells were then incubated with or without LPS (1 μg/ml) in the absence or presence of various concentrations of 2,4-bis(*p*-hydroxyphenyl)-2-butenal at 37°C for an additional 24 h. At that point, 10 μl of the WST-8 solution was added to the wells and the incubation was continued for another 1 h. The resulting color was assayed at 450 nm using a microplate absorbance reader (SunriseTM, TECAN, Switzerland).

### Determination of nitrite production

Astrocytes and microglial BV-2 cells were grown in 96-well plates and then incubated with or without LPS (1 μg/ml) in the absence or presence of 2,4-bis(*p*-hydroxyphenyl)-2-butenal at various concentrations for 24 h. Nitrite levels in the supernatant was assessed by Griess reaction [31]. Each 50 μl of culture supernatant was mixed with an equal volume of Griess reagent [0.1% N-(1-naphthyl)-ethylenediamine, 1% sulfanilamide in 5% phosphoric acid] and incubated at room temperature for 10 min. Absorbance at 540 nm was measured in a microplate absorbance reader, and a series of known concentrations of sodium nitrite was used as a standard.

### Measurement of ROS

Generation of ROS was assessed by 2,7-dichlorofluorescein diacetate (DCFH-DA, Sigma Aldrich), an oxidation-sensitive fluorescent probe. Intracellular H_2_O_2 _or low-molecular-weight peroxides can oxidize 2,7-dichlorofluorescein diacetate to the highly fluorescent compound dichlorofluorescein (DCF). Briefly, astrocytes were plated in 6-well plates (5 × 10^4^), and subconfluent cells were subsequently treated with 2,4-bis(*p*-hydroxyphenyl)-2-butenal (2.5-10 μg/ml) for 30 min. After the cells were trypsinized, 1 × 10^4 ^cells were plated in a black 96-well plate and incubated with 10 μM DCFH-DA at 37°C for 4 h. Fluorescence intensity of DCF was measured in a microplate-reader at an excitation wavelength of 485 nm and an emission wavelength of 538 nm.

### Measurement of PGE_2_

Cell media samples were analyzed for PGE2 with kits purchased from R&D Systems (Minneapolis, MN) according to manufacturer's instructions.

### Western blot analysis

To obtain total cell lysates, cells were homogenized with Protein Extraction Solution (PRO-PREPTM, Intron Biotechnology, Korea), and lysed by a 40 min incubation on ice. The lysate was centrifuged at 15,000 rpm for 15 min. To investigate protein expression in nuclear extract, cells were processed using a method for nuclear extract described in the section on electrophoretic mobility shift assay (EMSA) below. Equal amounts of proteins (40 μg) were separated on a SDS/10%-polyacrylamide gel, and then transferred to a polyvinylidene difluoride (PVDF) membrane (GE Water & Process technologies, Trevose, PA). Blots were blocked for 2 h at room temperature with 5% (w/v) non-fat dried milk in Tris-buffered saline Tween-20 [TBST: 10 mM Tris (pH 8.0) and a 150-mM NaCl solution containing 0.05% Tween-20]. After a short wash in TBST, membranes were incubated at room temperature with specific antibodies. Rabbit polyclonal antibodies against iNOS (1:1,000 dilution, Abcam) and COX-2 (1:1,000 dilution, Cayman Chemical, Ann Arbor, MI), APP (1:500 dilution, ABR, Golden, CO, USA), BACE1 (1:500 dilution, St. Louis, MO, USA), C99 (1:500 dilution, St. Louis, MO, USA), and rabbit polyclonal antibodies against p65 and IκBα (1:500 dilution), and mouse monoclonal antibody against p50 (1:500 dilution) (Santa Cruz Biotechnology Inc. Santa Cruz, CA) were used in study. Blots were then incubated with the corresponding conjugated anti-rabbit or mouse immunoglobulin G-horseradish peroxidase (Santa Cruz Biotechnology Inc. Santa Cruz, CA). Immunoreactive proteins were detected with an ECL western blotting detection system.

### Gel electromobility shift assay (EMSA)

Gel shift assays were performed according to the manufacturer's recommendations (Promega, Madison, WI). Briefly, 5 × 10^6 ^cells was washed twice with 1× PBS, followed by the addition of 1 ml of PBS, and the cells were transferred into cold Eppendorf tubes. The cells were spun down at 13,000 rpm for 5 min, and the resulting supernatant was removed. Cells were suspended in 400 μl of solution A containing 10 mM HEPES, pH 7.9, 1.5 mM MgCl_2_, 10 mM KCl, 0.5 mM dithiothreitol, 0.2 mM phenylmethylsulfonyl fluoride, and vigorously vortexed. Then, cells were allowed to incubate on ice for 10 min and centrifuged at 12,000 rpm for 6 min. The pelleted nuclei were resuspended in solution C (solution A + 420 mM NaCl, 20% glycerol) and allowed to incubate on ice for 20 min. The cells were centrifuged at 15,000 rpm for 15 min, and the resulting nuclear extract supernatant was collected in a chilled Eppendorf tube. Consensus oligonucleotides were end-labeled using T4 polynucleotide kinase and [γ-^32^P] ATP for 10 min at 37°C. Gel shift reactions were assembled and allowed to incubate at room temperature for 10 min followed by the addition of 1 μl (50,000-200,000 cpm) of ^32^P end-labeled oligonucleotide and another 20 min of incubation at room temperature. Subsequently 1 μl of gel loading buffer was added to each reaction and loaded onto a 6% nondenaturing gel and electrophoresis was performed until the dye was four-fifths of the way down the gel. Gels were dried at 80°C for 1 h and exposed to film overnight at -70°C.

### Transfection and assay of NF-κB luciferase activity

Astrocytes were plated at a density of 1 × 10^5 ^cells per 24-well plate. After 24 h of growth to 90% confluence, the cell were transfected with pNF-κB-Luc plasmid (5 × NF-κB; Stratagene, CA) using a mixture of plasmid and lipofectAMINE PLUS in OPTI-MEN according to manufacturer's specification (Invitrogen, Carlsbad, CA). Luciferase activity was measured by using a luciferase assay kit (Promega) according to the manufacturer's instructions (WinGlow, Bad Wildbad, Germany).

### Quantitative real-time PCR

For mRNA quantification, total RNA was extracted using an RNAqueous kit and cDNA was synthesized from 1 μg of total RNA using a High Capacity RNA-to-cDNA kit (Applied Biosystems, Foster City, CA) according to the manufacturer's protocol. Quantitative real-time PCR was performed using specific primers for GAPDH (Mm99999915_g1), IL-6 (Mm00446190_m1), TNF-α (Mm00443258_m1), IL-1β (Mm00434228_m1) in a 7500 Real-Time PCR System (Applied Biosystems). Thermocycling conditions include an initial denaturation of 20 s at 95°C, followed by 60 cycles of 95°C for 30 s and 60°C for 30 s). The values obtained for target gene expression were normalized to GAPDH and quantified relative to expression in control samples. For calculation of relative quantification, the 2-ΔΔCT formula was used, where -ΔΔCT = (CT,target-CT,GAPDH) experimental sample - (CT,target-CT,GAPDH) control sample.

### β- and γ- Secretase activities assay

The total activities of β- and γ-secretases (the protein preparation was same as the western blot) were determined using a commercially available β-secretase fluorescence resonance energy transfer (BACE 1 FRET) assay kit (PANVERA, Madison, USA) and a γ-secretase activity kit, (R&D systems, Wiesbaden, Germany), respectively, according to the manufactures' instructions. Each cell preparation was lysed in cold 1 × cell extraction buffer (a component of the kit) to a final protein concentration of 1 mg/ml. To determine β-secretase activity, 10 μl of lysate was mixed with 10 μl BACE1 substrate (Rh-EVNLDAEFK-Quencher), and then the reaction mixture was incubated for 1 h at room temperature in black 96-microwell plates. The reaction was stopped by adding 10 μl of BACE1 stop buffer (2.5 M sodium acetate). Fluorescence was determined using a Fluostar galaxy fluorometer (excitation at 545 nm and emission at 590 nm) equipped with Felix software (BMG Labtechnologies, Offenburg, Germany). Enzyme activity was linearly related to fluorescence increases, and activity is expressed as fluorescence units. All controls, blanks and samples were run in triplicate. To determine γ-secretase activity, 50 μl of lysate was mixed with 50 μl of reaction buffer. Next, the mixture was incubated for 1 h in the dark at 37°C after 5 μl of substrate was added. Substrate conjugated to the reporter molecules EDANS and DABCYL was cleaved by γ-secretase and released a fluorescent signal. This fluorescence was measured using a Fluostar galaxy fluorometer (excitation at 355 nm and emission at 510 nm) equipped with Felix software (BMG Labtechnologies, Offenburg, Germany). Levels of γ-secretase enzymatic activity were found to be proportional to fluorescence increases, and γ-secretase activity is expressed as fluorescence units.

### Fluorescence microscopy

The fixed cells were exposed to following primary antibodies; GFAP and Aβ_42 _(1: 100 dilutions in blocking serum, Abcam), Iba1 (1:100 dilution in blocking serum, Wako) at room temperature for 1 h. After incubation, the cells were washed twice with ice-cold PBS and incubated with an anti-rabbit or mouse secondary antibody conjugated to Alexa Fluor 488 or 568 (Invitrogen-Molecular Probes, Carlsbad, CA) at room temperature for 1 h. Immunofluorescence images were acquired using an inverted fluorescent microscope Zeiss Axiovert 200 M (Carl Zeiss, Thornwood, NY).

### Measurement of Aβ levels

Cell lysates (the same preparation of lysates as used for western blotting) were obtained using protein extraction buffer containing protease inhibitor, 4-(2-aminoethyl)-benzene sulfonyl fluoride. Aβ_42 _levels were determined using specific ELISAs (IBL, Immuno-Biological Laboratories Co., Ltd., Japan). In brief, 100 μl of sample was added to precoated plates and was incubated overnight at 4°C. After washing each well of the precoated plate with washing buffer, 100 μl of labeled antibody solution was added and the mixture was incubated for 1 h at 4°C in the dark. After washing, chromogen was added and the mixture was incubated for 30 min at room temperature in dark. Finally, the resulting color was assayed at 450 nm using a microplate absorbance reader (SunriseTM, TECAN, Switzerland) after addition of stop solution.

### Statistical evaluation

Data are presented as mean ± S.E. for three independent experiments performed in triplicate. Statistical analysis was performed by one-way ANOVA, followed by a Dunnett test as *post hoc *comparison. Differences were considered significant at *P < 0.05*.

## Results

### Effect of 2,4-bis(*p*-hydroxyphenyl)-2-butenal on astrocyte and microglial BV-2 cell viability

**Figure 2:**
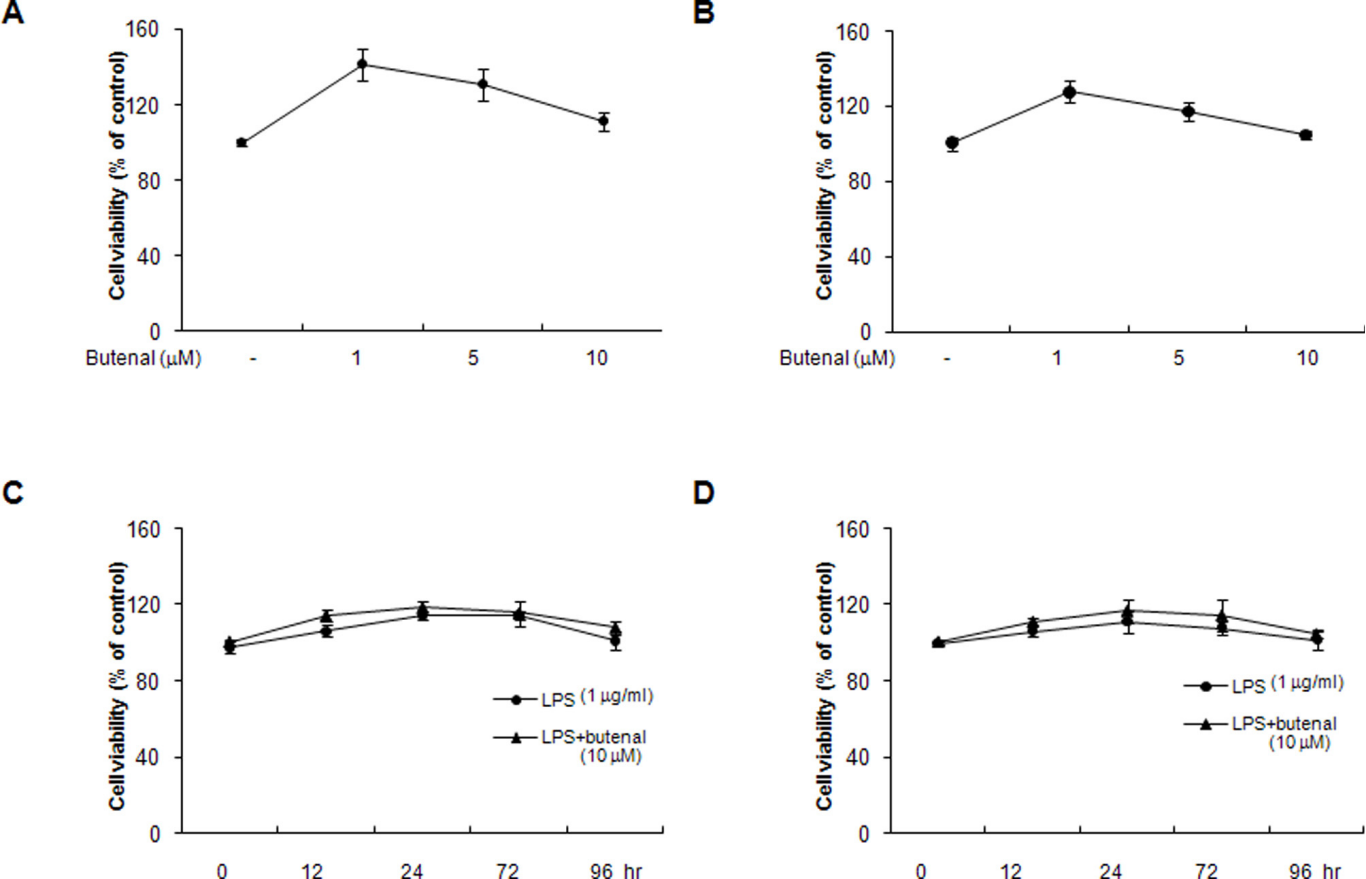
**Effect of 2,4-bis(*p*-hydroxyphenyl)-2-butenal on viability of astrocytes and microglial BV-2 cells**. Cell viability was evaluated using a WST-8 assay as described in Methods. Astrocytes (A) and microglial BV-2 cells (B) were incubated with 2,4-bis(*p*-hydroxyphenyl)-2-butenal (1-10 μM) in the absence of LPS for 72 h. Astrocytes (C) and microglial BV-2 cells (D) were incubated with 2,4-bis(*p*-hydroxyphenyl)-2-butenal (10 μM) in the presence of LPS (1 μg/ml) for 96 h. Results are given as a percentage of viable cells related to untreated controls. The data represent the mean ± S.E. for three independent experiments performed in triplicate.

Treatment of astrocytes and microglial BV-2 cells with 2,4-bis(*p*-hydroxyphenyl)-2-butenal resulted in a slight increase in cell viability at concentrations of 1 or 5 μM, and showed little reduction (< 20%) in cell viability at 10 μM concentration (Figure 2A and 2B). Furthermore, co-treatment with 10 μM 2,4-bis(*p*-hydroxyphenyl)-2-butenal and 1 μg/ml LPS did not shown significant difference in viability of astrocytes (Figure 2C) and microglial BV-2 cells (Figure 2D). Thus, in this study, anti-inflammatory and anti-amyloidogenesis effects were performed with less than 5 μM of 2,4-bis(*p*-hydroxyphenyl)-2-butenal.

### Effect of 2,4-bis(*p*-hydroxyphenyl)-2-butenal on LPS-induced ROS, NO, TNF-α, and interleukin (IL)-1β production in astrocytes and in microglial BV-2 cells

**Figure 3:**
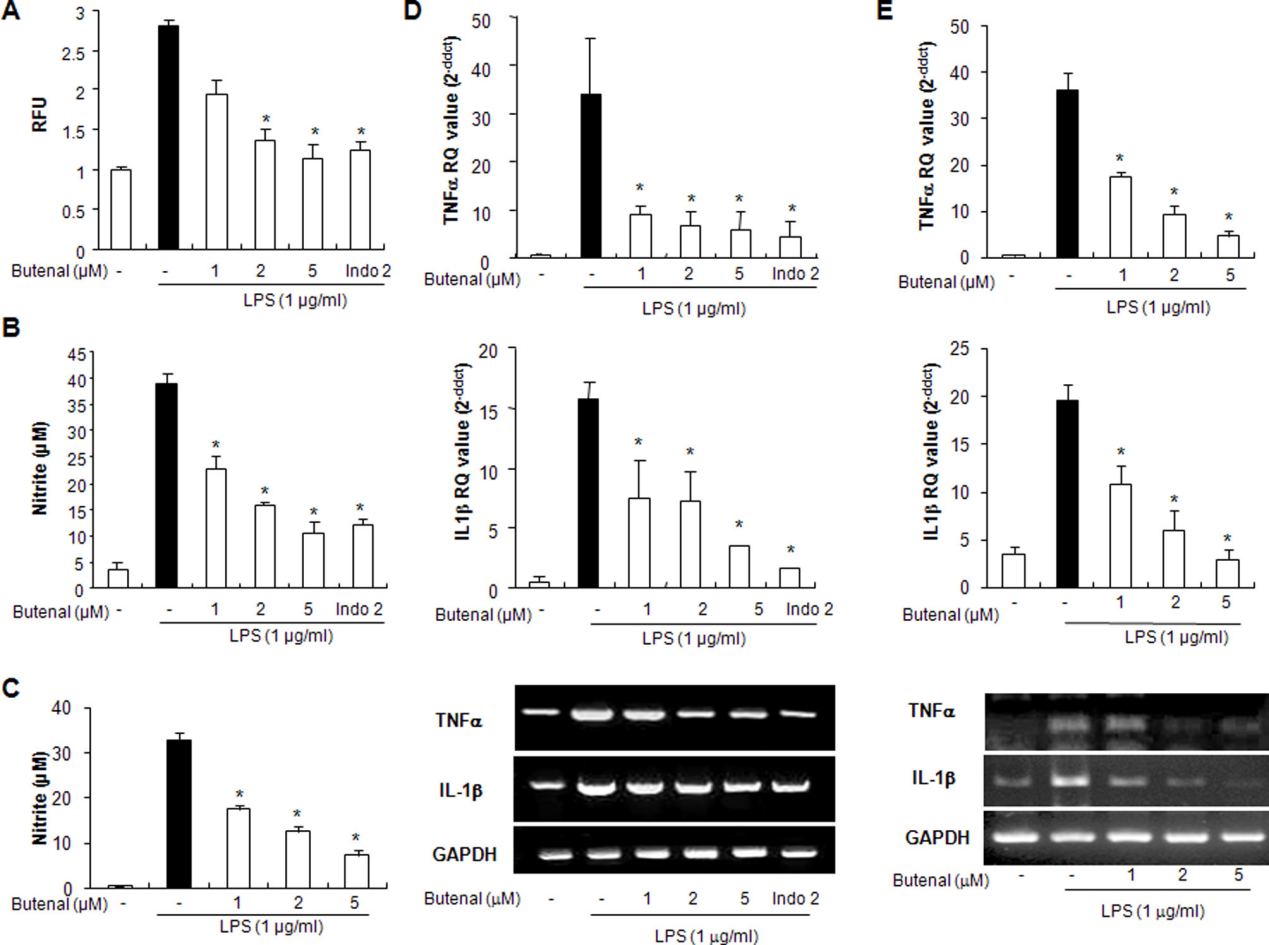
**Effect of 2,4-bis(*p*-hydroxyphenyl)-2-butenal on LPS-induced ROS and NO release, and expression of cytokines in astrocytes and microglial BV-2 cells**. Cells were treated with 1 μg/ml of LPS alone, or with LPS plus different concentrations (1, 2, 5 μM) of 2,4-bis(*p*-hydroxyphenyl)-2-butenal, at 37°C for 24 h. Intracellular ROS levels were determined by measuring DCF fluorescence (A). NO level was determined by Griess reaction, as described in Methods, in supernatants from astrocytes (B) and microglial BV-2 cells (C). mRNA levels for TNF-α and IL-1β were determined by real-time PCR, as described in Methods, in astrocytes (D) and microglial BV-2 cells (E). Values represent means ± SD for three independent experiments performed in triplicate. * indicates significantly different from LPS treated group (P < 0.05).

To study the protective effect of 2,4-bis(*p*-hydroxyphenyl)-2-butenal on LPS-induced activation of astrocytes and microglial BV-2 cells, the cells were treated with or without 2,4-bis(*p*-hydroxyphenyl)-2-butenal in the presence of LPS (1 μg/ml). Release of ROS and NO was determined as an indicator of astrocyte and microglial BV-2 cell activation as well as oxidative stress, and generation of TNF-α and IL-1β was measured as an indicator of astrocyte and of microglial BV-2 cell activation. We found that treatment with 2,4-bis(*p*-hydroxyphenyl)-2-butenal reduced LPS (5 μg/ml)-induced ROS in astrocytes (Figure 3A), and NO generation in astrocytes and microglial BV-2 cells (Figure 3B and 3C). TNF-α and IL-1β generation was also reduced in cultured astrocytes (Figure 3D) and microglial BV-2 cells (Figure 3E) in a concentration-dependent manner. Notably, these inhibitory effects of 2,4-bis(*p*-hydroxyphenyl)-2-butenal (5 μM) are comparable to the effects of indomethacin (2 μM), a well-known anti-inflammatory compound.

### Effect of 2,4-bis(*p*-hydroxyphenyl)-2-butenal on LPS-induced iNOS and COX-2 expression

**Figure 4:**
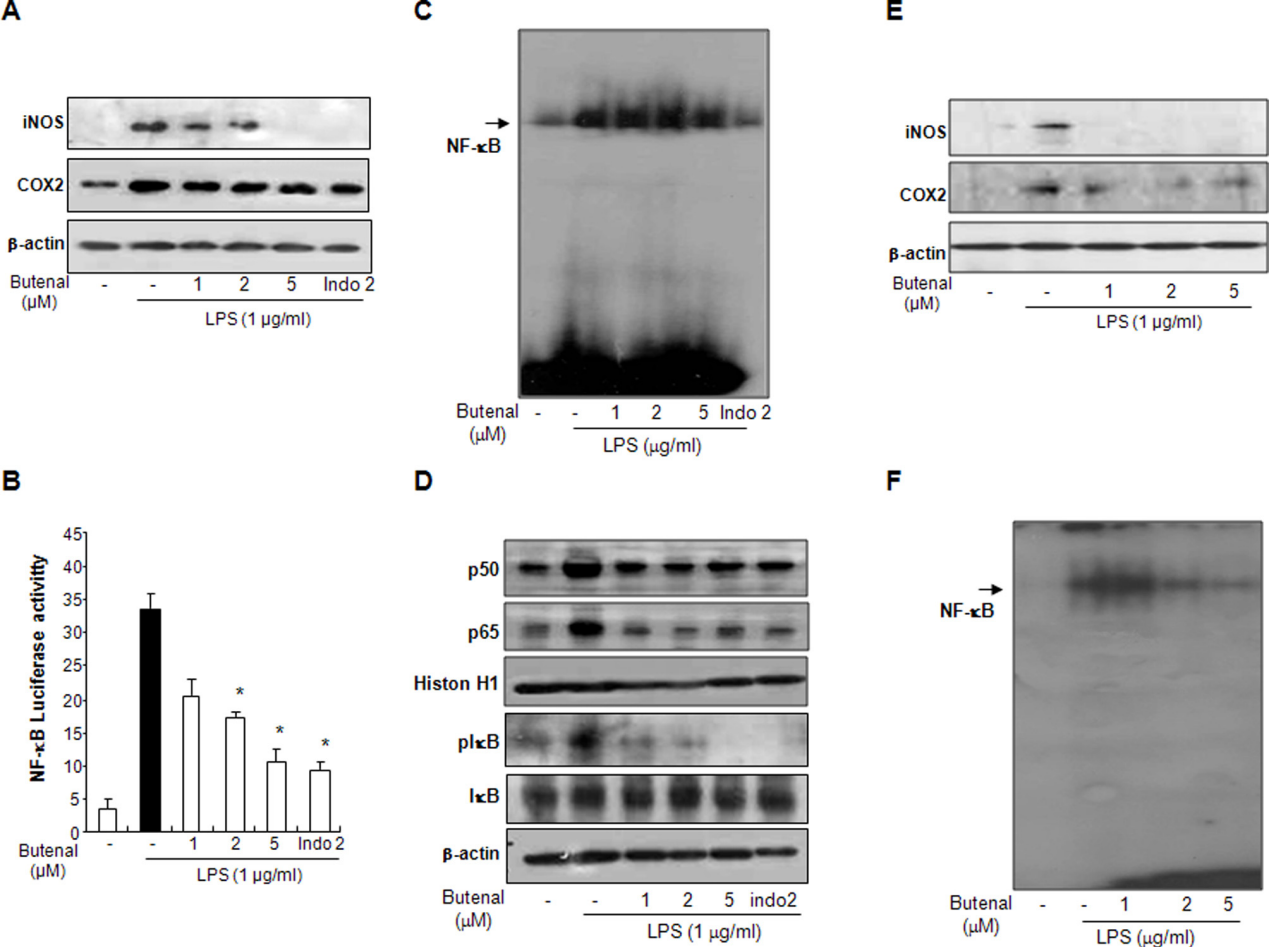
**Effects of 2,4-bis(*p*-hydroxyphenyl)-2-butenal on protein expressions of iNOS and COX-2, and on NF-κB-dependent luciferase activity and NF-κB DNA binding activity in astrocytes and microglial BV-2 cells**. Astrocytes (A) and microglial BV-2 cells (E) were treated with 1 μg/ml of LPS alone, or with LPS plus different concentrations (1, 5, 10 μM) of 2,4-bis(*p*-hydroxyphenyl)-2-butenal, at 37°C for 24 h. Equal amounts of total protein (40 μg/lane) were subjected to 10% SDS-PAGE, and the expression of iNOS and COX-2 were detected by western blotting using specific antibodies. β-Actin protein was used here as an internal control. Similar results were obtained from at least three different sets of experiment. Astrocytes were transfected with a p-NF-κB-Luc plasmid (5 × NF-κB), and then treated with LPS (1 μg/ml) either alone or with 2,4-bis(*p*-hydroxyphenyl)-2-butenal (1, 2, 5 μM) for 37°C for 8 h. Luciferase activity was then determined as described in Methods (B). Astrocytes (C) and microglial BV-2 cells (F) were treated with 1 μg/ml of LPS alone, or with LPS plus different concentrations (1, 5, 10 μM) of 2,4-bis(*p*-hydroxyphenyl)-2-butenal at 37°C for 1 h. Activation of NF-κB was investigated using EMSA as described in Methods. Nuclear extracts from astrocytes treated with LPS alone (1 μg/ml) or with 2,4-bis(*p*-hydroxyphenyl)-2-butenal (1, 2, 5 μM) and LPS were subjected to DNA binding reaction with ^32^P end-labeled oligonucleotide specific to NF-κB. Specific DNA binding of the NF-κB complex is indicated by an arrow. Similar results were obtained from at least three different sets of experiments. Equal amounts of nuclear extract (40 μg) were subjected to 10% SDS-PAGE. Nuclear translocation of p50 and p65, and degradation of IκB were detected by western blotting using specific antibodies. β-Actin protein was used here as an internal control (D). Values represent the mean ± S.E. for three independent experiments performed in triplicate, and each luciferase activity was calibrated using the amount of protein. * indicates significantly different from the LPS-treated group (P < 0.05).

Because iNOS can also be modulated by COX-2, we investigated whether the inhibitory effect of 2,4-bis(*p*-hydroxyphenyl)-2-butenal on astrocyte and microglial BV-2 cell activation and NO production occurs via inhibition of iNOS and COX-2 gene expression, using western blot analysis, In agreement with a resting astrocytic state of non-activation, un-stimulated cells expressed extremely low levels of iNOS and COX-2 protein. However, iNOS and COX-2 protein expression was markedly increased in response to LPS (1 μg/ml) after 24 h. Treatment with 2,4-bis(*p*-hydroxyphenyl)-2-butenal (1, 5, 10 μM) caused a concentration-dependent decreases in LPS-induced iNOS expression in astrocytes (Figure 4A). This result is consistent with the inhibitory profile of 2,4-bis(*p*-hydroxyphenyl)-2-butenal on NO production. A similar inhibitory effect of 2,4-bis(*p*-hydroxyphenyl)-2-butenal on LPS-induced COX-2 expression was also observed in astrocytes (Figure 4A).

### Effect of 2,4-bis(*p*-hydroxyphenyl)-2-butenal on LPS-induced NF-κB luciferase and DNA-binding activities

NF-κB controls the expression of mRNA for iNOS and COX-2, whose products contribute to the pathogenesis of inflammatory processes. To investigate whether 2,4-bis(*p*-hydroxyphenyl)-2-butenal is able to attenuate LPS-induced NF-κB-mediated promoter activity, we used a luciferase reporter gene expressed under the control of 5 κB *cis*-acting elements. Astrocytes were transiently transfected with the NF-κB-dependent luciferase reporter construct according to the manufacturer's specifications (Invitrogen). Cells were then treated with LPS (1 μg/ml) or co-treated with LPS and 2,4-bis(*p*-hydroxyphenyl)-2-butenal for 8 h. Treatment with 2,4-bis(*p*-hydroxyphenyl)-2-butenal resulted in a concentration-dependent suppression of luciferase activity induced by LPS (Figure 4B). To investigate whether 2,4-bis(*p*-hydroxyphenyl)-2-butenal can also inhibit NF-κB DNA-binding activity, astrocytes were co-treated with LPS and 2,4-bis(*p*-hydroxyphenyl)-2-butenal for 60 min, this being the time after which maximal activation of NF-κB is observed following LPS treatment (data not shown). Nuclear extracts from co-treated cells were prepared and assayed for NF-κB DNA-binding by EMSA. LPS induced strong NF-κB binding activity in cultured astrocytes, which was inhibited by co-treatment with 2,4-bis(*p*-hydroxyphenyl)-2-butenal (Figure 4C).

To elucidate the mechanism of inhibition of 2,4-bis(*p*-hydroxyphenyl)-2-butenal on LPS-induced NF-κB, translocation of p50 and p65 as well as IκBα degradation were examined. 2,4-Bis(*p*-hydroxyphenyl)-2-butenal prevented the LPS-induced increase in nuclear translocation of p50 and p65 in astrocytes, in a concentration-dependent manner. 2,4-Bis(*p*-hydroxyphenyl)-2-butenal inhibited the LPS-induced degradation of IκBα (Figure 4D). Moreover, we also found that 2,4-bis(*p*-hydroxyphenyl)-2-butenal prevented LPS-induced iNOS and COX-2 expression (Figure 4E) as well as NF-κB binding activity (Figure 4F) in microglial BV-2 cells. These results indicate that 2,4-bis(*p*-hydroxyphenyl)-2-butenal inhibits LPS-induced activation of NF-κB via inhibition of both IκBα degradation as well as p50 and p65 translocation into the nucleus. These inhibitory effects of 2,4-bis(*p*-hydroxyphenyl)-2-butenal on NF-κB activity, and iNOS and COX-2 expression, are also comparable to the effects of indomethacin (2 μM).

### 2,4-Bis(*p*-hydroxyphenyl)-2-butenal prevents LPS-induced amyloidogenesis

**Figure 5:**
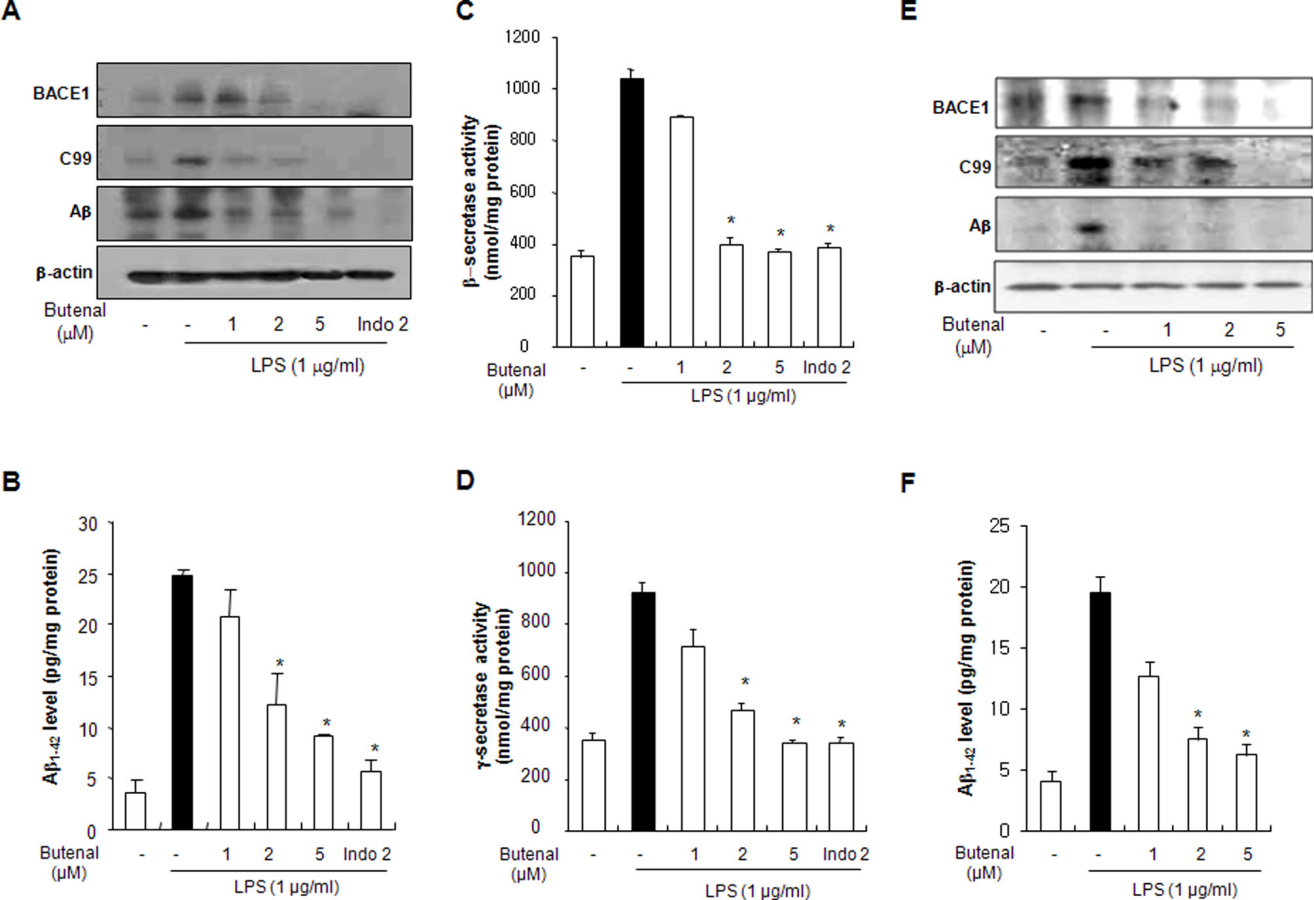
**Effect of 2,4-bis(*p*-hydroxyphenyl)-2-butenal on expression of BACE1, C99 and Aβ**_**42**_**, secretase activity and Aβ**_**42 **_**level. Expressions of BACE1, Aβ**_**42 **_**and C99 were detected by western blotting using specific antibodies in astrocytes (A) and microglial BV-2 cells (E)**. Each blot is representative of three experiments. β-Actin protein was used here as an internal control. *p < 0.05 indicates significantly different from the LPS-treated group. Co-treatments with 2,4-bis(*p*-hydroxyphenyl)-2-butenal and LPS for 24 h were used. Media were collected to determine Aβ_42 _secretion by ELISA from astrocytes (B) and microglial BV-2 cells (F). Values represent the mean ± S.E. of three independent experiments with triplicate. The activities of β-secretase and of γ-secretase were assessed using commercially available assay kits as described in Methods (C and D). Values represent mean ± S.E. for three independent experiments performed in triplicate. * indicates significantly different from LPS treated group (p < 0.05).

**Figure 6:**
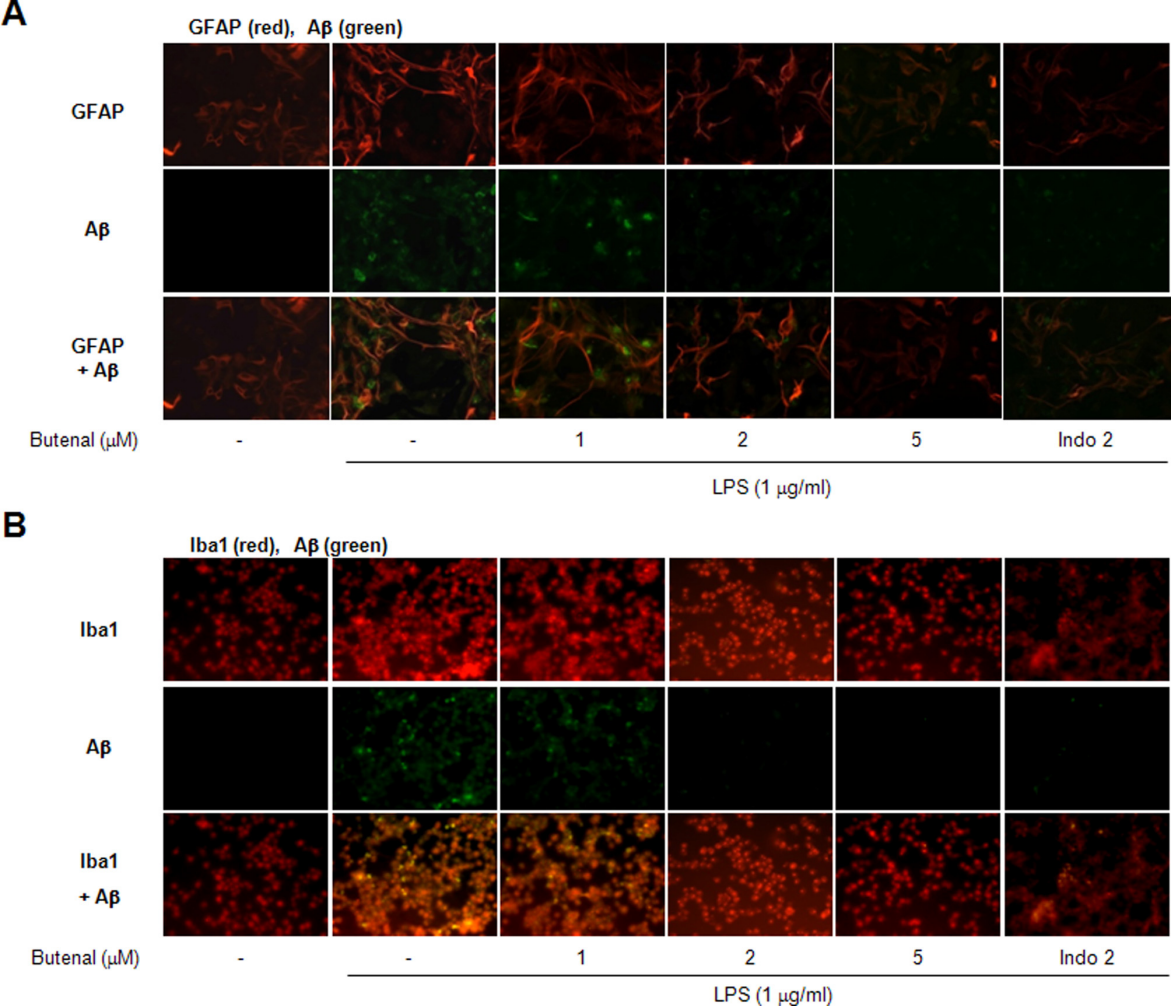
**Expression of activation markers (GFAP for astrocytes and Iba1 for microglia) and Aβ**_**42 **_**in astrocytes and in microglial BV-2 cells, observed by double-fluorescence**. Confocal microscope observation was performed as described under in Methods. Cultured astrocytes were incubated with anti-GFAP and anti-Aβ_42 _primary antibodies (A), and the microglial BV-2 cells were incubated with anti-Iba1 and anti-Aβ_42 _primary antibodies (B). Fluorescence was developed using Alexa 568-conjugated anti-rabbit and Alexa 488-conjugated anti-mouse secondary antibodies. Images of astrocytes double-labeled with GFAP (red) and Aβ_42 _(green) antibodies show the fluorescent antibody staining separately and merged. Images of microglial BV-2 cells double-labeled with Iba1 (red) and Aβ_42 _(green) antibodies show the fluorescent antibody staining separately and merged.

The effect of inflammation on amyloidogenesis *in vitro *was also investigated, because neuro-inflammation can cause amyloid generation, and microglia and astrocytes are a major source of neuro-inflammation. Astrocytes and microglia lend both mechanical and metabolic support to neurons, regulating the environment in which they function. To determine the relationship between neuro-inflammation and amyloidogenesis, we investigated whether the anti-inflammatory effect of 2,4-bis(*p*-hydroxyphenyl)-2-butenal could result in anti-amyloidogenesis. As shown in Figure 5A, when unstimulated, the cells expressed low levels of APP, β-site APP cleavage enzyme (BACE) and C99 protein, whereas the expressions of BACE and C99 proteins increased in response to LPS (1 μg/ml) after 24 h. In addition, 2,4-bis(*p*-hydroxyphenyl)-2-butenal also decreased LPS-induced Aβ_42 _secretion into the culture media of astrocytes (Figure 5B). Consistent with the expression of these proteins, activation of β- and γ-secretases, which are the rate-limiting enzymes in Aβ generation, was also increased by LPS, but inhibited by 2,4-bis(*p*-hydroxyphenyl)-2-butenal in a concentration-dependent manner (Figure 5C and 5D). In microglial BV-2 cells, we also found that 2,4-bis(*p*-hydroxyphenyl)-2-butenal inhibited LPS-induced expression of BACE1, C99 and Aβ (Figure 5E) as well as Aβ level in a concentration-dependent manner (Figure 5F). Since activation of astrocytes is implicated in the activation of β-secretase, we investigated whether the numbers of activated (GFAP-positive) astrocytes and accumulation of Aβ (Aβ_42_-postive cells) were concomitantly increased by LPS, and whether 2,4-bis(*p*-hydroxyphenyl)-2-butenal would reduce astrocyte activation, thereby reducing Aβ-levels. To demonstrate this more clearly, cells immunoreactive for both GFAP and Aβ_42 _were identified using a double immunofluorescence method. The co-reactive cell number for both markers was markedly increased by LPS, but was lowered by 2,4-bis(*p*-hydroxyphenyl)-2-butenal treatment (Figure 6A). Moreover, to determine if treatment by 2,4-bis(*p*-hydroxyphenyl)-2-butenal inhibits LPS-induced amyloidogenesis, we investigated the effects of 2,4-bis(*p*-hydroxyphenyl)-2-butenal in another neuroglial cell type, microglial BV-2 cells. The co-reactive cell number for both activation of microglia (Iba1-postive cells) and Aβ accumulation (Aβ_42_-positive cells) was increased by LPS, but was lowered by 2,4-bis(*p*-hydroxyphenyl)-2-butenal treatment (Figure 6B). These results further indicate that the amyloidogenic pathway can be promoted by neuro-inflammatory stimulation, and the anti-inflammatory effect of 2,4-bis(*p*-hydroxyphenyl)-2-butenal can result in anti-amyloidogenesis.

### Effect of 2,4-bis(*p*-hydroxyphenyl)-2-butenal on STAT3 activities

**Figure 7:**
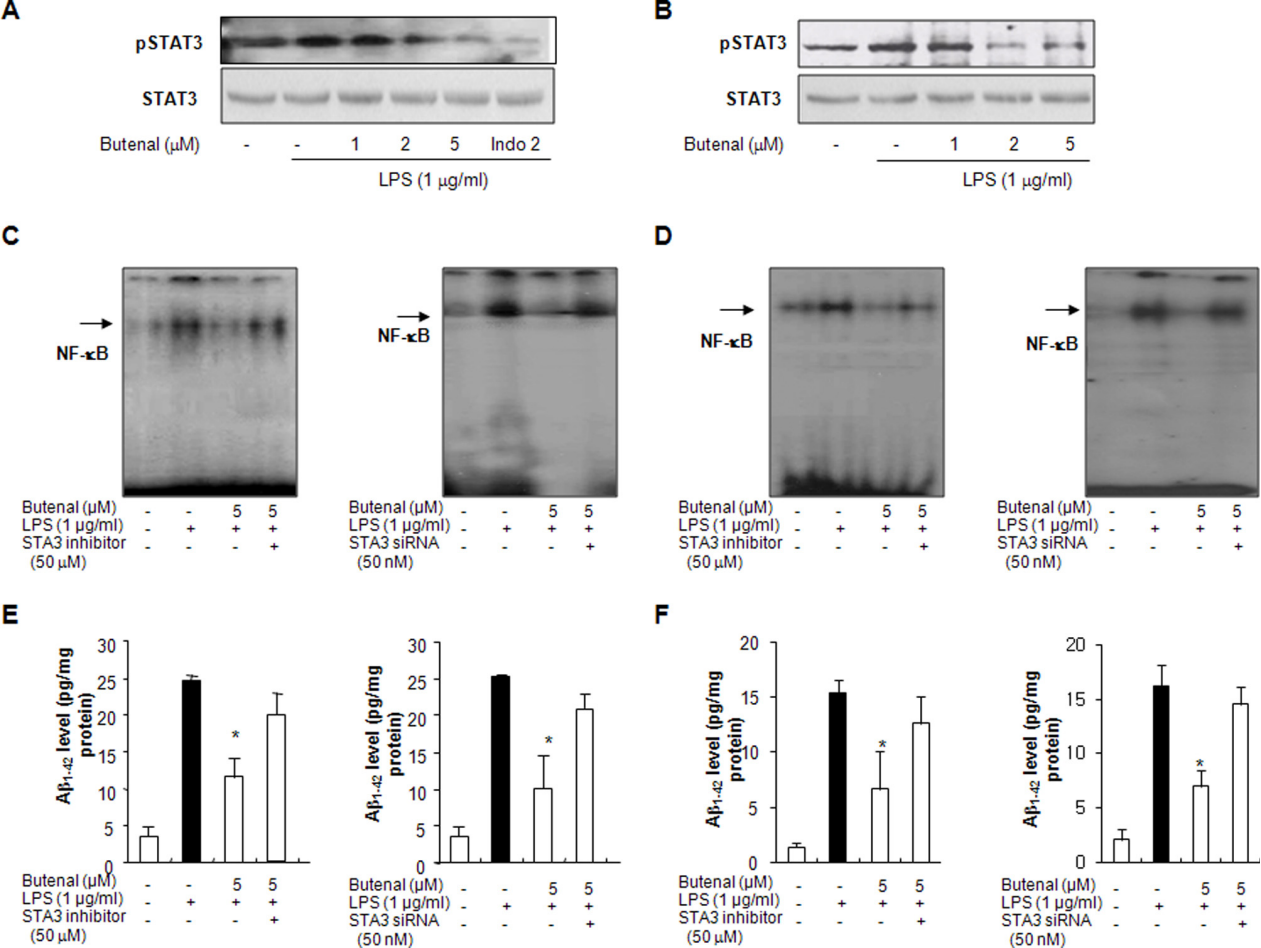
**Effects of 2,4-bis(*p*-hydroxyphenyl)-2-butenal on STAT3 activation in cultured astrocytes and in microglial BV-2 cells**. The cells were treated with 1 μg/ml of LPS alone, or with LPS plus different concentrations (1, 5, 10 μM) of 2,4-bis(*p*-hydroxyphenyl)-2-butenal at 37°C for 1 h. Equal amounts of total proteins (40 μg/lane) were subjected to 10% SDS-PAGE, and activation of STAT3 (phosphorylation) was detected by western blotting using specific antibodies in astrocytes (A) and in microglial BV-2 cells (B). To block the STAT3 pathway, cells were treated with the STAT3 inhibitor AG490 (50 μM) or with 50 nM siRNA for STAT3 for 1 h prior to treatment with 2,4-bis(*p*-hydroxyphenyl)-2-butenal. Effect of 2,4-bis(*p*-hydroxyphenyl)-2-butenal on NF-κB DNA binding activity in astrocytes (C) and microglial BV-2 cells (D), and Aβ_42 _level in astrocytes (E) and microglial BV-2 cells (F). For determination of Aβ_1-42 _levels, cells were treated with 1 μg/ml of LPS alone, or with LPS plus different concentrations (1, 5, 10 μM) of 2,4-bis(*p*-hydroxyphenyl)-2-butenal at 37°C for 24 h. Values are mean ± S.E. for three experiments performed in triplicate. * indicates significantly different from LPS treated group (P < 0.05).

STAT3 cooperates with NF-κB in controlling the expression of genes contributing to inflammatory processes, as well as amyloidogenesis. To investigate whether 2,4-bis(*p*-hydroxyphenyl)-2-butenal can prevent LPS-induced STAT3 activity, astrocytes and microglial BV-2 cells were treated with LPS (1 μg/ml) or co-treated with LPS and 2,4-bis(*p*-hydroxyphenyl)-2-butenal for 24 h. LPS-induced STAT3 activity (phosphorylation), which was markedly inhibited by co-treatment with 2,4-bis(*p*-hydroxyphenyl)-2-butenal in astrocytes and microglial BV-2 cells in a concentration-dependent manner (Figure 7A and 7B).

### Involvement of the STAT3 pathway in the inhibitory effect of 2,4-bis(*p*-hydroxyphenyl)-2-butenal on LPS-induced neuroinflammation and amyloidogenesis

To further examine the mechanisms regulating neuroinflammation and amyloidogenesis by STAT3 and NF-κB, we used siRNA and a pharmacological inhibitor of STAT3 in astrocytes and microglial BV-2 cells activated by LPS, and investigated the participation of the STAT3 pathway in neuroinflammation and amyloidogenesis. 2,4-bis(*p*-hydroxyphenyl)-2-butenal inhibited Aβ production and NF-κB activity induced by LPS treatment in astrocytes. These inhibitory effects were abolished by down-regulation of STAT3 expression with siRNA and with the pharmacological STAT3-specific inhibitor AG490 (50 μM) in cultured astrocytes (Figure 7C and 7E) and microglial BV-2 cells (Figure 7D and 7F). These findings indicate that activation of STAT3 by 2,4-bis(*p*-hydroxyphenyl)-2-butenal may not only inhibit neuroinflammation but also prevent neuro-inflammation-induced Aβ production in astrocytes and microglial BV-2 cells.

## Discussion

Epidemiological and genetic evidence has shown that an inflammatory process contributes to AD pathology. We have previously shown that there is a convincing link between neuro-inflammatory reactions and amyloidogenesis, and that anti-inflammatory agents prevent neuroinflammation as well as amyloidogenesis [9,32,33]. In the present *in vitro *study, we confirmed that genes involved in inflammation and amyloidogenesis are concomitantly increased by LPS treatment; however, 2,4-bis(*p*-hydroxyphenyl)-2-butenal prevented LPS-induced neuroinflammation and amyloidogenesis. These anti-inflammatory and anti-amyloidogenic effects may be related to inhibition of NF-κB and STAT3 activity by 2,4-bis(*p*-hydroxyphenyl)-2-butenal. Consequently, the results presented in this study indicate that 2,4-bis(*p*-hydroxyphenyl)-2-butenal could be useful for the treatment and/or prevention of AD through its anti-neuroinflammatory properties.

By means of *in vivo *studies, we and others had recently showed that LPS can influence Aβ deposition [8,9] and that anti-inflammatory agents prevent Aβ deposition [11,33]. Ibuprofen, a commonly used, non-steroidal anti-inflammatory drug (NSAID), reduces Aβ levels, Aβ burden, and brain inflammation in a mouse model of AD (Tg2576) [34], and indomethacin, given to Tg2576 mice, also reduces insoluble Aβ_40 _and Aβ_42 _levels in hippocampus [12]. Ibuprofen also decreases cytokine-stimulated Aβ production in human neuronal cells and astrocytes [10]. The present data show that severe neuroinflammation, as evaluated by COX-2 and iNOS expression, and expression of BACE and C99 proteins are increased in astrocytes in response to LPS. However, 2,4-bis(*p*-hydroxyphenyl)-2-butenal decreased expression of BACE and C99, as well as Aβ_42 _secretion, in LPS-stimulated cells. These 2,4-bis(*p*-hydroxyphenyl)-2-butenal-induced reversals of LPS-induced changes were accompanied by reductions in gene expressions for iNOS and COX-2, and consequent decreases in NO and ROS production, as well as decreased IL-1β and TNF-α generation. It is noteworthy that TNF- α has been shown to increase production of Aβ [35]. We have also reported co-elevated expression of Aβ_42 _and COX-2, as well as of TNF-α and IL-1β, in presenilin 2-mutant AD transgenic mice, and these expressions are prevented by an anti-inflammatory compound, 4-*O*-methylhonokiol [32]. Our present results suggest that 2,4-bis(*p*-hydroxyphenyl)-2-butenal also has anti-neuroinflammatory effects, and that these effects result in inhibition of amyloidogenesis induced by LPS.

The mechanism by which 2,4-bis(*p*-hydroxyphenyl)-2-butenal prevents amyloidogenesis is unclear. Because APP is first cleaved by β-secretase at its β-cleavage site, generating membrane-bound C99 whose subsequent proteolysis by γ-secretase produces Aβ_42_, we examined the effect of 2,4-bis(*p*-hydroxyphenyl)-2-butenal in the absence or presence of LPS on β- and γ- secretase activity. Consistent with the effects on Aβ_42 _generation, as well as BACE and C99 expression, LPS-induced increases in β- and γ-secretase activities were prevented by 2,4-bis(*p*-hydroxyphenyl)-2-butenal. The inflammatory cytokines IL-1β, IL-6, TNF-α and TGF-β have been shown to augment APP expression [36,37] and Aβ formation [35], and these processes may be related to the activation of transcriptional upregulation of β-secretase mRNA, protein and enzymatic activity [7]. TNF-α, IL-1β and IFN-γ stimulate γ-secretase and consequently control Aβ generation [38]. Rogers et al. [39] have shown that primary inflammatory cytokines enhance APP gene expression at the transcriptional level through the well-characterized IL-1-responsive element of APP mRNA. Thus, LPS-enhanced inflammatory cytokines could influence APP processing and/or secretase activity (enhancement of β- and γ-secretases), thereby affecting amyloidogenesis. These findings suggest that one mechanism by which 2,4-bis(*p*-hydroxyphenyl)-2-butenal prevents amyloidogenesis is due, in part, to a decrease in LPS-induced production of inflammatory cytokines, which may activate β- and γ-secretases.

**Figure 8:**
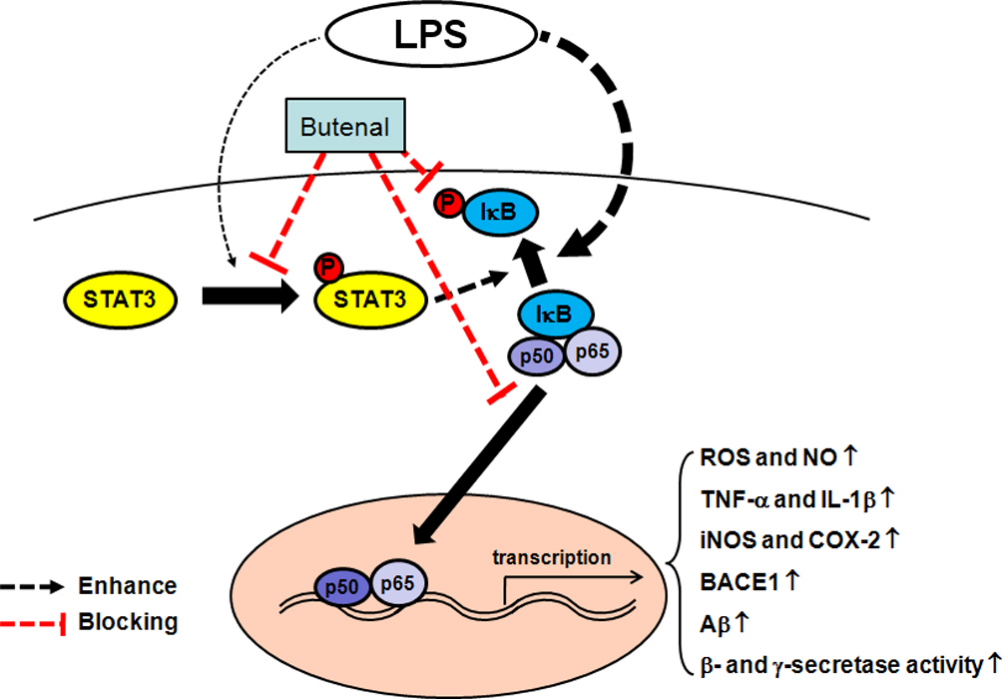
**Schematic representation of a functional pathway of 2,4-bis(*p*-hydroxyphenyl)-2-butenal action**. 2,4-Bis(*p*-hydroxyphenyl)-2-butenal blocks NF-κB- and STAT3-mediated upregulation of amyloidogenesis and neuro-inflammation induced by LPS.

Even though cytokine reduction, following 2,4-bis(*p*-hydroxyphenyl)-2-butenal treatment could influence secretases involved in amyloidogenesis induced by LPS, the signaling involved is poorly understood. It is noteworthy that the APP and BASE1 genes promoters contain a potential NF-κB recognition site [40-46], thus activation of NF-κB, an important transcription factor involved in the expression of many inflammatory genes induced by LPS, could also directly affect APP promoter activity. Therefore, the inhibition of NF-κB by 2,4-bis(*p*-hydroxyphenyl)-2-butenal could play a significant role in reduction of amyloidogenesis, as well as neuroinflammation induced by LPS. It is also important to note that IL-6-family cytokines increase the expressions of IL-1β, TNF-α and COX-2, and markedly increase phosphorylation of STAT3 in primary cultures of rat microglia [47]. Several studies have shown that STATs have been involved in amyloidogenesis through the modulation of secretases. Cho et al. showed that IFNγ-induced BACE1 expression is mediated by activation of the JAK2 signaling pathway, an upstream pathway of STATs, and by direct binding of STAT1 to the BACE1 promoter in astrocytes [5]. In our study, 2,4-bis(*p*-hydroxyphenyl)-2-butenal-induced inhibition of LPS-induced Aβ production and NF-κB activity in astrocytes and microglial BV-2 cells was abolished by down-regulation of STAT3 expression with siRNA and with the pharmacological STAT3-specific inhibitor AG490. These findings indicate that activation of STAT3 by 2,4-bis(*p*-hydroxyphenyl)-2-butenal may not only inhibit neuroinflammation but also prevent neuro-inflammation-induced Aβ production in cultured astrocytes and microglial BV-2 cells. In addition, blocking NF-κB by 2,4-bis(*p*-hydroxyphenyl)-2-butenal is critical for the neuroinflammatory and amyloidogenic effects of 2,4-bis(*p*-hydroxyphenyl)-2-butenal. Moreover, blocking STAT3 abolished the inhibitory effect of 2,4-bis(*p*-hydroxyphenyl)-2-butenal on NF-κB and amyloidogenesis induced by LPS. These results suggest that STAT3-dependent NF-κB inactivation may be a critical signal in the anti-neuroinflammation and anti-amyloidogenesis of 2,4-bis(*p*-hydroxyphenyl)-2-butenal as seen in Figure 8.

Inflammatory processes play a critical role in the pathogenesis of many human diseases. Macrophage overproduction of inflammatory mediators such as cytokine and NO, for example, have also been implicated in neuroinflammatory diseases such as AD [48]. Neuroglia, including astrocytes and microglia, play a pivotal role in regulating aspects of inflammation in the central nervous system. A convincing link between the neuro-inflammatory reaction and amyloidogenesis has been well demonstrated, and anti-inflammatory agents can concomitantly prevent neuroinflammation as well as amyloidogenesis. Our present data indicate that 2,4-bis(*p*-hydroxyphenyl)-2-butenal, a new product from a tyrosine-fructose Maillard reaction could be useful for treatment and/or prevention of neuroinflammatory diseases such as AD.

## Competing interests

The authors declare that they have no competing interests.

## Authors' contributions

Hong JT designed the study and prepared the manuscript. Lee YJ, Choi DY and Choi IS performed experiments. Jeong HS isolated and characterized 2,4-bis(*p*-hydroxyphenyl)-2-butenal. Han SB, Oh KW and Han JY discussed the study. All authors have read and approved the final version of this manuscript.
